# Pneumatic conveying printing technique for bioprinting applications†

**DOI:** 10.1039/c9ra07521f

**Published:** 2019-12-11

**Authors:** Izabella Brand, Isabel Groß, Dege Li, Yanzhen Zhang, Anja U. Bräuer

**Affiliations:** Carl von Ossietzky University of Oldenburg, Faculty of Mathematics and Science, Department of Chemistry D-26111 Oldenburg Germany zhangyanzhen.upc@gmail.com; Research Group Anatomy, School for Medicine and Health Science, Carl von Ossietzky University Oldenburg Oldenburg Germany anja.braeuer@uni-oldenburg.de; China University of Petroleum, College of Mechanical and Electronic Engineering 266580 Qingdao China; Research Center for Neurosensory Science, Carl von Ossietzky, University Oldenburg Oldenburg Germany

## Abstract

Droplet-based bio-printing (DBB) techniques have been extensively accepted due to their simplicity, flexibility and cost performance. However, the applicability of inkjet printing for bioprinting techniques still faces challenges, such as a narrow range of available bio-ink materials, cell damage due to the pressure strike and high shear rate during the printing process. Here, a new droplet-based printing technique, pneumatic conveying printing (PCP), is described. This new technique is successfully adopted for cell-printing purposes. The cells present in the bio-ink are not exposed to any significant pressure and therefore the PCP technique is gentle to the cells. Furthermore, PCP allows the usage of inks with viscosities higher than 1000 mPa s, enabling the usage of bio-inks with high cell concentrations (several tens of millions per millilitre). As a proof of concept, two different cell types were printed with this novel technique. To achieve a printing resolution of 400 to 600 μm, cells were encapsulated into a hydrogel containing calcium alginate. Deposition of the bio-ink drop containing sodium alginate on a surface pre-treated in CaCl_2_ solution, ensures a fast cross-linking reaction and the formation of gel drops. Cells encapsulated in the alginate gel survive and proliferate. Our novel PCP technique is highly suitable for 2D and 3D cell bio-printing.

## Abstract 2

Droplet-based bio-printing (DBB) techniques have been extensively accepted due to their simplicity, flexibility and cost performance.

## Graphical abstract

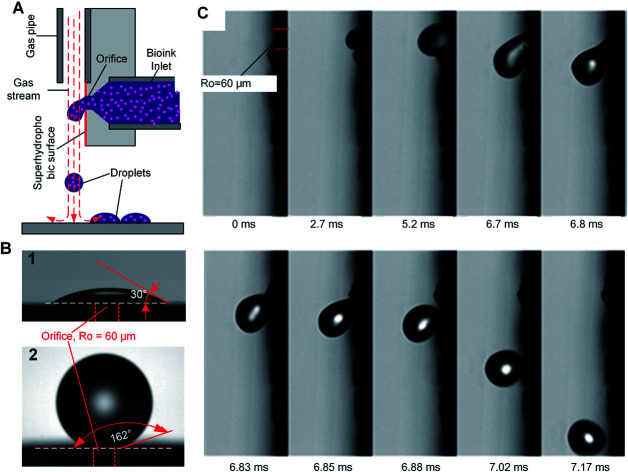

## Introduction

Bioprinting is a new and rapidly growing scientific discipline that applies design principles to biological building blocks, such as cells and biomolecules.^1–15^ The purpose of this sophisticated combination is the delivery of a product that will help to solve urgent problems in regeneration medicine, cancer research, drug development, bio-preservation, and the food industry. The current development in the 2D and 3D printing technologies makes feasible the achievement of this challenging aim. Indeed, bioprinting technology has already exhibited the ability to fabricate some simple tissue analog structures, such as implantable bone tissue,^16–18^ skin tissues,^19^ heart tissue,^10,20,21^ vascular networks,^11,22^ or a bionic ear.^15^ However, 3D bioprinting is still in a rudimentary state. There is still a long way to go to achieve the ultimate goal of fabricating functional replacement human organs for use in the clinic.^7,9,12^

One of the stumbling blocks that hinder the development of 3D bioprinting is the lack of suitable bio-ink delivery techniques.^23–25^ Due to the vulnerability of the biological materials (*e.g.* cells, tissues), bioprinting has distinct requirements compared to conventional printing techniques.^6,24–27^ First, the ink-delivery methods should be as gentle as possible, to avoid damage to the biological material in the bio-ink. Second, the bio-ink should be printed on a surface (or bulk) where the biomimetic environment preserves the activity of the printed biological material. Normally, printing has to be conducted in a sterile environment with constant humidity and temperature.

The existing bioprinting techniques are divided into three categories: droplet-based,^6,28–30^ extrusion-based,^31,32^ and laser-based techniques^33^ (ESI 1†). Droplet-based bioprinting (DBB) methods were the first used in the field of cell printing, tissue engineering and organ fabrication.^1,6,7,29^ The existence of a large number of DBB methods points to their wide use in the bioprinting technology. Compared to extrusion-based and laser-assisted bioprinting techniques, DBB offers greater advantages due to its simplicity, flexibility, and precise control of the deposition of biological material.^6,25^ In DBB techniques, the bio-ink is ejected *via* an orifice either by the high pressure inside the ink chamber, for example in inkjet- and micro-valve printing,^30^ or by an external force generated by sound^28^ or high voltage.^6,27^ Depending on the drop-dispensing mechanisms, inkjet printing can be classified into thermal-bubble inkjets and piezoelectric inkjets. For the thermal inkjet, the pressure increases due to a rapid expansion of a bubble generated by the transient evaporation of the ink by a thermal–electrical resistor. Whereas for the piezoelectric inkjet, the drops are ejected by a quick form change of a micro-piezo ceramic attached to the ink chamber, which thereby reduces the volume of the chamber. The largest drawback of inkjet-based bioprinting is the extremely high pressure strike in the chamber, the exceptionally high shear rate at the orifice, due to its small radius (several tens of micrometers), and the high ejection speed (several m s^−1^). These parameters reduce the cells' viability. Another drawback of the inkjet printing is its incapability of handling viscous inks.^6,25^ To ensure printability, the viscosity of bio-inks should be lower than 20 mPa s.^6,34^ Unfortunately, most of the bio-inks have viscosity higher than this. On the one hand the bio-ink formulation depends on the selected cells, biomaterial on which the cells are printed, and is specific for each cell assembly or engineered tissue.^8,13,24,35–37^ It brings the need of an individual adjustment of the bio-ink composition. On the other hand bio-inks should have common characteristics: low viscosity, biodegradability, biocompatibility, enhanced adhering properties and high mechanical strength. Various strategies in the bio-ink formulation have been used to achieve print confidence and required resolution. In other words, the cells printed on the biomaterial surface should remain their physiological activity.

The printing of cells directly on surfaces mimicking the extracellular matrix often leads to complete wetting of the surface by the bio-ink and loss of the printing resolution.^2^ To increase the printing resolution and provide biomimetic environment for cells, the hydrogels are often used in the bioprinting technology.^8,36,38^ Suitable hydrogels are characterized either by a fast gelling rate or fast crosslinking mechanism.^38^ In addition, they are biocompatible to a given cell type, have short-time stability, are bio-degradable in a long-time scale, promote cell–cell interactions, proliferation and functions. Natural (*e.g.* collagen, fibrin, hyaluronic acid, hydroxyapatite, alginate or chitosan), synthetic (*e.g.* polylysine, polylactic acid or polyethylene glycol), and hybrid (mixture of natural and synthetic) hydrogels are used for in the bio-ink formulation.^36,38,39^ For example, a bio-ink containing sodium alginate sol, in contact with solution containing divalent cations undergoes immediate gelation.^31,40,41^ Alginic acid is a polysaccharide with homopolymeric blocks of (1–4)-linked β-d-mannuronate (M) and a-l-guluronic acid (G) forming regions of M-(MM), G-(GG), and alternating (GM) structures. In the presence of divalent cations (*e.g.* Ca^2+^) the carboxylate residues in sodium alginate become immediately crosslinked forming a hydrogel. In the presence of ligands such as EDTA, anions precipitating calcium salts (*e.g.* PO_4_^3−^, CO_3_^2−^) or acidic solutions the stability of the alginate gel decreases. Due to a fast gelation rate and slow decomposition of the gel in the presence of ligands or ions interacting strongly with the Ca^2+^ ions, alginate hydrogel has large application potential in bioprinting technology.

In this paper, we introduce a new drop-on-demand inkjet technique: pneumatic conveying printing (PCP), which is almost pressure-free during the printing process and is capable of handling viscous bio-inks. To improve the printing resolution of the PCP technique sodium alginate is added to the bio-ink solution. Bio-inks with high concentration of cells (6 ×10^6^ cells per mL) are printed on glass surfaces modified with Matrigel matrix which were pre-treated with CaCl_2_ solution.

## Results and discussion

### Principle of PCP technique

**Fig. 1:**
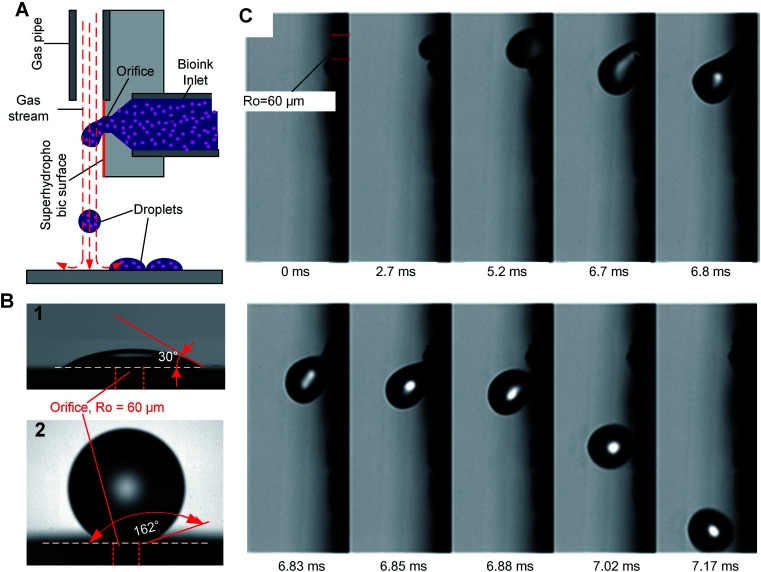
Pneumatic conveying printing. (A) Principle of PCP; (B) comparison of drops shape on hydrophilic and hydrophobic surface; (C) high speed image of the drop generation and conveying process of PCP.

We introduce a new drop-on-demand inkjet technique: pneumatic conveying printing (PCP). It is almost pressure-free during the printing process and is capable of handling viscous bio-inks. The scheme illustrating the principle of the new pneumatic conveying printing technique is shown in Fig. 1A. The bio-ink is extruded from a small orifice that opens at the superhydrophobic surface of the printing nozzle. The axis of the orifice is oriented parallel to the printed surface. This construction differs from all currently used bioprinting techniques: inkjet printing, electrohydrodynamic-jet printing, and extrusion-based printing, in which the axis of the orifice is perpendicular to the printed surfaces (ESI 2†). The superhydrophobic surface is the key to implementing PCP. On a hydrophilic surface (Fig. 1B1) the ink will spread due to the large adhesion force between the ink drop and the surface. In this case, the adhesion force is too large for the droplets to be blown off by the air stream. On a super-hydrophobic surface, the adhesion force of a hydrophilic drop is negligible and the contact area is minimal. As shown in Fig. 1B2, the contact angle of micro droplets (*R* = 300 μm) on the super-hydrophobic surface is 162°. Photographs in Fig. 1C show high-speed images of drop generation in the PCP. On the superhydrophobic surface, the ink squeezed from the orifice adopts a spherical shape and, once the Stocks' force generated by the gas stream overcomes its adhesion force, the neck connecting the drop and the orifice breaks and the droplet detaches from the surface. Subsequently it is carried to the printing surface by the gas stream. Fig. 1C shows that *ca.* 7 ms are required to produce a drop of the bio-ink, indicating that the printing frequency is high (*ca.* 140 Hz). Extrusion of aqueous liquids from super hydrophobic nozzle^42^ or oleic/organic liquids from superamphiphobic nozzle^43^ had been recently reported. However, after extrusion the transfer of the pendulous droplet was achieved by contacting the substrate or just by the action of the gravity.^42,43^ Therefore, the printing frequency is very limited. In our case, a gas stream was introduced to accelerate the detachment process and higher printing frequency can be achieved.

**Fig. 2:**
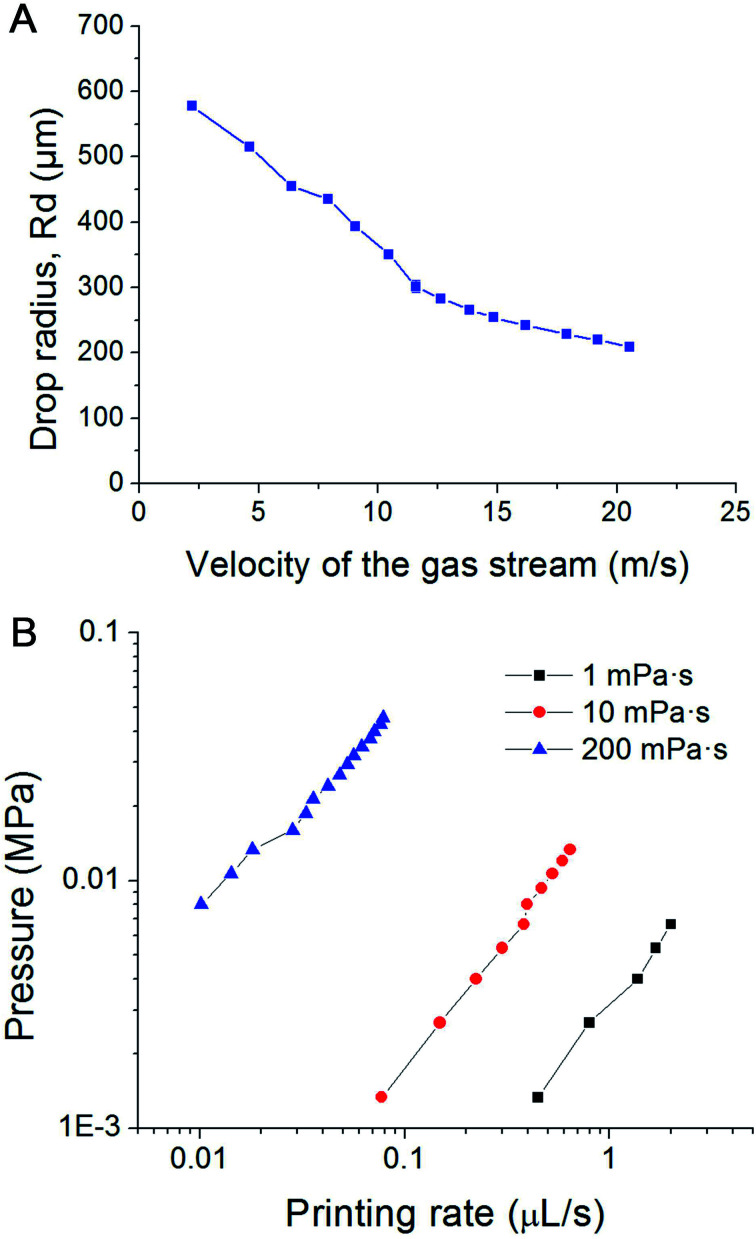
(A) Influence of the velocity of the air stream on the size of the droplets; (B) pressure within the pipeline filled with inks of different viscosity *vs.* printing rate plots in PCP.

In our experiments, the radius of the orifice is *ca.* 100 μm. The distance between the superhydrophobic surface and the ink chamber is small (∼100 to 200 μm). This constructional solution leads to a significant reduction of the flow resistance and pressure inside the whole pipeline. Moreover, it avoids orifice clogging to a large extent. The ink extrusion is controlled by a programmed micropump that can extrude the ink with an adjustable flow rate. During printing, the distance between the substrate and nozzle is close to 5 mm. The bio-ink flows continuously from the nozzle at a constant rate (8.2 μL s^−1^ in described below in the experimental part). A drop of liquid is extruded from the nozzle on the hydrophobic surface of the printer. At the distance of 100–200 μm above the nozzle a gas pipe is mounted. The diameter of the gas pipe is similar to that of the orifice (100–160 μm). It is important that the gas pipe and ink pipe are perpendicular to each other. It is connected to a gas reservoir by a flow-rate regulating device. The air stream will generate a downward drag force applied on the drop, free drop will be generated once the drag force overcome the capillary force of the neck (the moments of 6.85 ms in Fig. 1C). The influence of flow velocity of the gas stream and ink viscosity on the size of the drops is shown in Fig. 2A. The drop size is determined by the velocity of the gas stream which in turn is determined by the gas pressure. The inks extruded from the nozzle is cut by the stream of gas and transported onto a substrate. The drop size decrease with increasing velocity of the gas stream, this can be explained by the stronger drag force generated by the higher velocity air stream.

### Bioprinting performance of PCP technique

During the whole printing process, no significant pressure is applied to the cell-laden inks. Depending on the size of the orifice, flow rate and viscosity of the ink, the velocity of the gas stream, and the printing frequency can be varied between several Hz and several hundred Hz. Furthermore, inks with a viscosity of about 1000 mPa s can be printed, greatly expanding the variety of printable inks. Fig. 2B shows plots of the liquid pressure in the pipeline, which arises from the flow resistance of the ink. For given geometry of the nozzle, the pressure in the pipe line is manily determined by the flow rate and viscosity of the ink. For a bio-ink with a low viscosity (*e.g.* 1 mPa s), the required pressure of the liquid is below 0.01 MPa, and thus lower than human blood pressure. For ink with high viscosity (*e.g.* 200 mPa s), the required pressure is between 0.01 to 0.1 MPa, comparable to atmospheric pressure. This pressure is harmless for most biological materials, including cells. Due to the low extrusion rate (8.2 μL s^−1^ in our experiments), the shear rate at the orifice is in the range of 0.003–0.7 s^−1^. This is about one million times smaller than the maximum shear rate of traditional inkjet printing.

The air stream will generate a downward drag force applied on the drop, free drop will be generated once the drag force overcome the capillary force of the neck (the moments of 6.85 ms in Fig. 1C). The influence of flow velocity of the gas stream and ink viscosity on the size of the drops is shown in Fig. 2B. The drop size is determined by the velocity of the gas stream which in turn is determined by the gas pressure. The inks extruded from the nozzle is cut by the stream of gas and transported onto a substrate. The drop size decrease with increasing velocity of the gas stream, this can be explained by the stronger drag force generated by the higher velocity air stream.

**Fig. 3:**
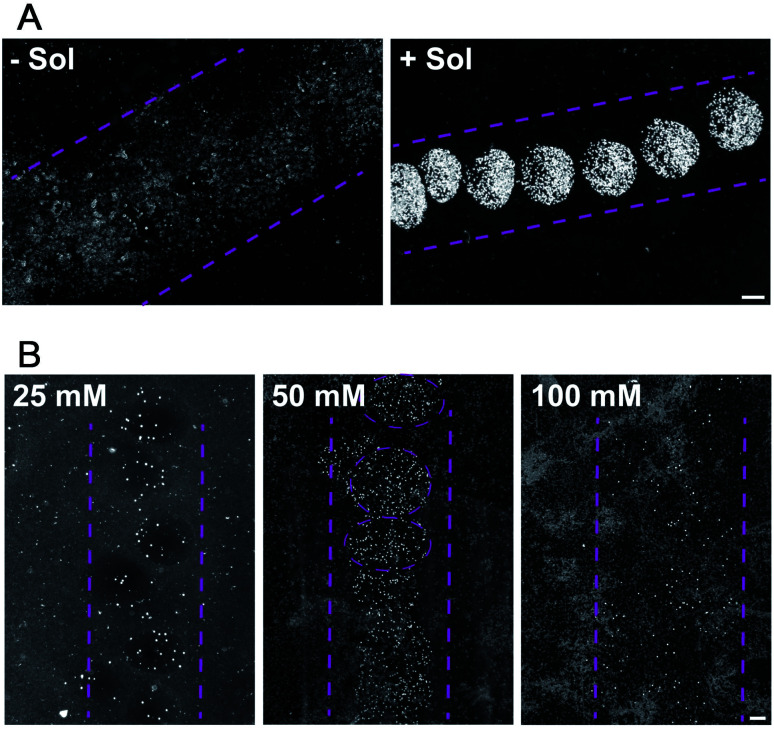
PCP with alginate bio-ink (Sol) on Matrigel-coated surfaces. (A) Comparison of the printing resolution using bio-ink without (−Sol) or with alginate (+Sol)-containing HAP1 cells on Matrigel-coated microscope slides; (B) comparison of printing alginate bio-ink containing HEK293H cells on Matrigel supplemented with different CaCl_2_ concentrations. Scale bars 200 μm.

For droplet based bioprinting, the printed line width is mainly dependent on the size of the drops and the wetting interaction between the drop and the substrate. Therefore, the composition of the bio-ink and properties of the surface, on which the drops spread, have large influence on the resolution of the printing technique. In the absence of sodium alginate in the HAP1 cells bio-ink solution, the resolution of the printing is very low. The drops of bio-ink spread on the surface and merge to form *ca.* 1500 μm wide line (Fig. 3A, −Sol). The addition of sodium alginate to the bio-ink solution enables a fast crosslinking of the biopolymer with the Ca^2+^ ions present on the Matrigel surface. The printing of a bio-ink containing 10 mg mL^−1^ of sodium alginate and HAP1 cells form a nozzle with a diameter of 160 μm results in a formation of drops with the average diameter of 471 ± 32 μm (Fig. 3A, +Sol).

Furthermore, we found that the Ca^2+^ ion concentration in the solution in which the printed substrate is incubated has a direct effect on the printed line width (Fig. 3B). At low (20 mM) Ca^2+^ concentration in the incubation solution, and thus at a lower content of Ca^2+^ ions on the Matrigel surface, droplets containing HEK293 cells in alginate gel are only weakly attached to the surface. The alginate sol is not sufficiently cross-linked with the surface Ca^2+^ ions and, therefore, weakly attached drops of variable size are printed on the Matrigel surface (Fig. 3B). An increase in the concentration of Ca^2+^ ions to 50 mM in the incubation solution results, during the printing, in an immediate formation of a cross-linked gel on the surface. Well-defined, stable gel drops with the average diameter of 650 ± 44 μm are formed on the Matrigel surface (Fig. 3B). A further increase in the concentration of the Ca^2+^ ions in the pre-incubation solution leads to a decrease in the resolution of the printing procedure. At high content of Ca^2+^ ions on the surface, the gel-formation process leads to cross-linking of the entire drop and affects the drop spreading on the surface (Fig. 3B). Moreover, an excess of Ca^2+^ ions on the surface reacts with phosphate and carbonate ions present in the electrolyte solution. This leads to the formation of precipitates on the surface, lowering the printing resolution. Thus the 50 mM concentration of CaCl_2_ is optimal to ensure a droplet-shaped printing pattern with a line resolution of 400–600 μm when compared to pipetted cells. Our first results demonstrate that the resolution and accuracy of the PCP depend on the following parameters:

(i) Bio-ink composition;

(ii) Bio-ink viscosity;

(iii) Preparation and modification of the substrate on which the bio-ink is printed and

(iv) To the less extend on the gas pressure.

Incubation of the glass substrate modified by the Matrigel film in CaCl_2_ solution seems to yield a non-uniform distribution of the Ca^2+^ ions on the substrate surface. The gelation process of sodium alginate strongly depends on the surface concentration of the Ca^2+^ ions. A non-uniform surface concentration of the Ca^2+^ ions affects the size of the gel drop as well as its position on the printed surface. Moreover, any variation of the pressure in the gas pipe may influence the accuracy of the printing technique. Therefore, a particular attention has to be kept to improve these factors to enable more general application of PCP in the bioprinting technology.

**Fig. 4:**
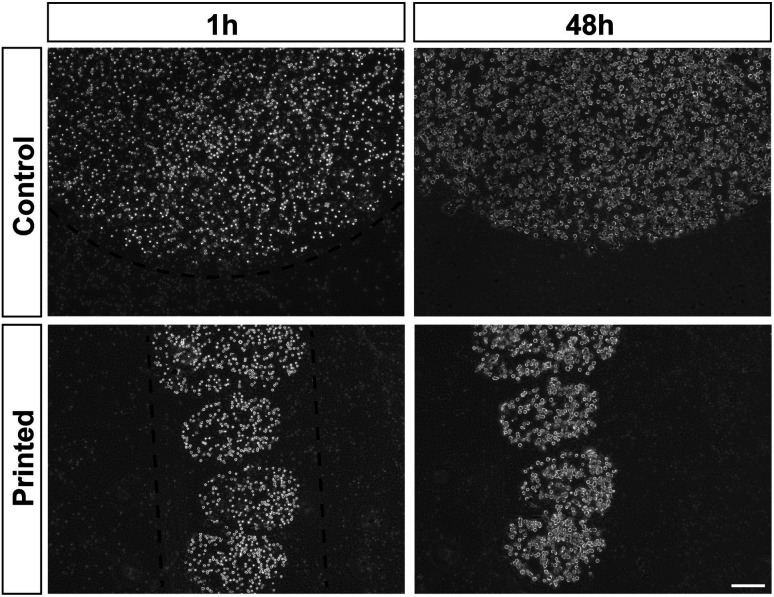
Proliferation of HEK293H cells after PCP in alginate bio-ink. HEK293H cells were printed or pipetted (Control) in alginate bio-ink on Matrigel-coated microscope slides and further cultured. Pictures taken after 1 h and 48 h. Scale bar 200 μm.

**Fig. 5:**
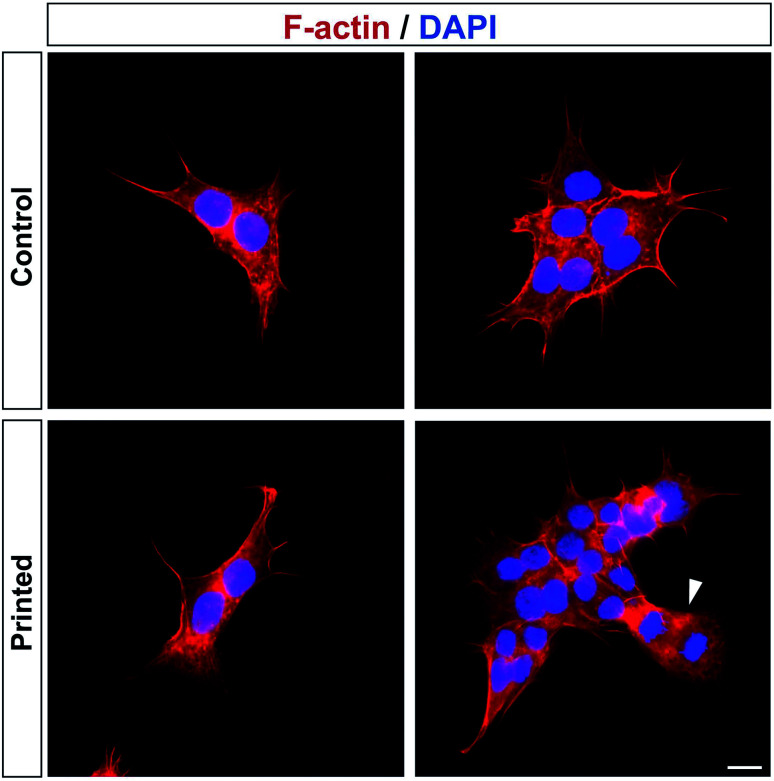
HAP1 cells after PCP. Immunostaining of cytoskeletal F-actin (red) and cell nuclei (blue) of HAP1 cells 4 days after PCP (Printed) and under control conditions (Control). The white arrow shows dividing cells. Scale bar 10 μm.

Cell division was monitored after printing and the results are shown in Fig. 4. This printing technique allows similar cell division as in controls (Fig. 4). All of the cells hold their position once printed on the surface. The printed-cell survival rate was obtained by comparing with the control, and a survival rate of almost 100% was achieved. No apparent difference of proliferation could be detected between the printed cell and the control. No stress fibers were detectable in printed HAP1 cells compared to unprinted HAP1 control cells, as shown by F-actin staining (Fig. 5). Immunostaining results of the printed HAP1 cells also exhibited the excellent health of the printed cells, as indicated by cell division (Fig. 5). No obvious differences could be detected between the printed cell and the control.

## Conclusions

In this work, a pneumatic conveying printing (PCP) technique was first introduced into the bioprinting field. The printing mechanism of PCP is distinct from traditional inkjet printing techniques. The droplets of PCP are generated by the shear action of the air stream, and the droplet-conveying process also can be performed by the same stream. During the pneumatic conveying printing, the pressure inside the pipeline is close to one standard atmospheric pressure and there is no significant pressure fluctuation. In contrast, the shear rate at the orifice is six orders of magnitude smaller than in existing inkjet-printing techniques. Thanks to the low pressure and shear-rate magnitude, mechanical damage to the cells can be greatly ameliorated, as indicated by their excellent cell health after printing. Another advantage of PCP is its capability of handling viscous bio-inks. Viscous bio-inks that cannot be printed by the traditional inkjet printing technique. However, they can be printed by PCP, demonstrating its potential to revolutionize bioprinting technology. PCP technique offers a new harmless for cell printing approach, which is applicable to different cell types. The challenge in the printing technology is the bio-ink formulation which will results in a fast formation of biocompatible, biodegradable, cell-containing gel drops in the printed assembly.

## Materials and methods

### Fabrication of the nozzle

The nozzle was homemade from a 40 × 10 × 2 (length × width × thickness) mm^3^ Teflon plate. First, a blind hole with diameter 1 mm was drilled on the back side of Teflon plate. The distance between the bottom of this blind hole and the front surface of the plate (on which the orifice opens) was held at about 150 μm. The orifice was made by impaling the blind hole with a micro needle. The Teflon surface, on which the orifice opens, was coated using commercially available coating (Never Wet, Tor Coatings Lim., Durham, UK) to achieve a super-hydrophobic surface. During the coating, a continuous flow of gas through the orifice prevented its blocking by the coating. A 32 G Stainless tube, with inner diameter 0.11 mm (B. Braun Melsungen AG, Melsungen, Germany) was used as the gas pipe and amounted directly above the orifice. Before assembly, all parts were washed in ethanol and placed in the clean lab.

### Printing operation

During printing, the bio-ink was supplied by a computer-controlled syringe pump with a resolution of 83 nL (GeSIM XP3000, GESIM, Dresden, Germany). The flow rate of the syringe pump could be adjusted between 0.4 and 62 μL s^−1^. Gas (Ar or CO_2_) was supplied by a high-pressure gas cylinder and the flow rate adjusted by a pressure-regulating valve. The pressure in the pipe during printing was measured by a precision pressure gauge (MicroFab, CT-PT-21, USA). Ca. 1 mL of the bio-ink solution was introduced into a syringe pump. Printing was carried out at the speed of 0.4 μL s^−1^. Lines were printed using 8.3 μL of the bio-ink per 7 cm line. These experiments were repeated three times. For control experiments, the same volume (8.3 μL) of freshly prepared bio-ink was pipetted onto Matrigel-covered microscope slides. Fully supplemented medium was added 30 minutes after printing, and cells were further cultured at 37 °C and 5% CO_2_ atmosphere.

### High speed photography

Observations of the dynamic formation and movement of droplets was carried out on the platform of an inverted optical microscope (Leica, DM i8, Germany). The whole process was monitored and recorded by a high-speed camera (Photron, SA-Z, Japan) connected to the optical microscope. The applied frame rates of this high-speed camera could be varied from 50 to 100 000 frames per second, with constant resolutions from 1024 × 1024 to 572 × 260 pixels.

### Cell culture, preparation of the bio-ink and substrates

HAP1 cells (Horizon Discovery, Waterbeach, UK, Catalog ID C631) were routinely maintained in Iscove's Modified Dulbecco's Medium (IMDM, Thermo Fisher Scientific, Waltham, MA, USA) supplemented with 10% fetal bovine serum (FBS, PAN-Biotech, Aidenbach, Germany), 100 units per mL penicillin and 100 μg mL^−1^ streptomycin (PAN-Biotech). HEK293H cells (Thermo Fisher Scientific, Catalog ID 11631017) were routinely maintained in Dulbecco's Modified Eagle Medium (DMEM, Thermo Fisher Scientific) supplemented with 10% FBS, 2 mM l-glutamine (Merck, Darmstadt, Germany), 100 units per mL penicillin and 100 μg mL^−1^ streptomycin. Both cell lines were cultured under standard conditions of 5% CO_2_ and 37 °C.

For printing experiments, microscopic glass slides were coated with 500 mg mL^−1^ Matrigel matrix (Corning, Corning, NY, USA) in IMDM or DMEM fully supplemented as described above and DMEM with additional 25, 50 or 100 mM CaCl_2_. Matrigel was allowed to solidify for at least 24 h at 37 °C. For immunohistochemical staining, glass coverslips were coated with 0.2 mg mL^−1^ poly-l-lysine (Merck, Steinheim, Germany) for 2 h at 37 °C.

Sodium alginate solution was prepared at a concentration of 10 mg mL^−1^ alginic acid sodium salt (Merck, Darmstadt, Germany) in IMDM with 10 mM EDTA or DMEM without calcium, both without any supplements mentioned above, and stirred for 2 h at room temperature. For sterilization, the alginate solution was introduced to UV light for 1 h.

For bio-ink preparation, cells were washed once with 1× phosphate-buffered saline (PBS, Thermo Fisher Scientific) and detached by a 5 min 0.05% trypsin–EDTA (Thermo Fisher Scientific) incubation at 37 °C. Trypsinization was stopped by the addition of fully supplemented media and cells were centrifuged for 5 min at 300 g. Supernatant was removed and the cell pellet was re-suspended in either IMDM for HAP1 cells or DMEM without calcium for HEK293H cells. Six million cells per mL were added to the prepared alginate solution. Depending on the bio-ink formula, the viscosity of the bio-inks varied from about 20 mPa s to about 50 mPa s.

### Immunohistochemistry and microscopy

For immunhistochemical staining of HAP1 cells, culture media was aspirated and cells were washed once with 1× PBS. Cells were fixed with ice-cold 4% paraformaldehyde in 1× PBS containing 15% sucrose for 20 min at room temperature (RT) and subsequently permeabilized with 0.1% Triton X-100 in 1× PBS for 3 min at 4 °C. For F-actin staining, Phalloidin-iFluor 594 (Abcam, Cambridge, United Kingdom) was diluted 1 : 1000 in 1% bovine serum albumin (Carl Roth, Karlsruhe, Germany) in 1× PBS and for staining of cell nuclei 0.5 μg mL^−1^ DAPI (Carl Roth) was added. Cells were incubated for 1 h at RT, washed with 1× PBS and coverslips were mounted on microscope slides with Shandon Immu-Mount mounting media (Thermo Fisher Scientific).

Bright-field images of printed HAP1 and HEK293H cells were captured with a CKX53 inverted microscope with integrated Phase Contrast (iPC) and a XM10 monochrome camera (Olympus, Shinjuku, Japan). Fluorescence images were acquired with an IX83 inverted imaging system with a DP80 camera (Olympus) and a 4-channel high-specification LED System (Judges Scientific, London, United Kingdom). Olympus cellSense software was used with both microscopes, and adjustments of brightness and contrast were carried out with ImageJ (NIH, Bethesda, MD, USA).

## Author contributions

I. B., Y. Z. and A. B. designed the experiments. I. B., I. G., and Y. Z. carried out the experiments. I. G., and A. B. contributed to the bio-ink preparation and cell characterization after printing. D. L., and Y. Z. contributed to the design and fabrication of the nozzle, high-speed imaging of the printing process- and CFD simulation studies.

## Conflicts of interest

There are no conflicts to declare.

## Supplementary Material

RA-009-C9RA07521F-s001
