# Supplementary material for: Pneumatic conveying printing technique for bioprinting applications
Source: RSC Adv. 2019 Dec 11;9(70):40910–6. doi: 10.1039/c9ra07521f (PMC9076373; doi:10.1039/c9ra07521f)
Supplement: RA-009-C9RA07521F-s001 [file RA-009-C9RA07521F-s001.pdf]

Electronic Supplementary Material (ESI) for RSC Advances.
This journal is © The Royal Society of Chemistry 2019

Pneumatic conveying printing technique for bioprinting applications

Izabella Brand[1], Isabel Groß[2], Dege Li[3], Yanzhen Zhang[1]*, Anja U. Bräuer[2,4]*

1. Carl von Ossietzky University of Oldenburg, Faculty of Mathematics and Science, Department of Chemistry, D-26111 Oldenburg, Germany

2. Research Group Anatomy, School for Medicine and Health Science, Carl von Ossietzky University Oldenburg, Oldenburg, Germany

3. China University of Petroleum, College of Mechanical and Electronic Engineering, 266580, Qingdao, China

4. Research Center for Neurosensory Science, Carl von Ossietzky, University Oldenburg, Oldenburg, Germany

Corresponding authors: Dr. Yanzhen Zhang, email: zhangyanzhen.upc@gmail.com; Prof. Dr. Anja U. Bräuer, email: anja.braeuer@uni-oldenburg.de

Supporting information

SI.1 Classification of the bioprinting techniques …………………..…………………………2

SI.2 Limitation of existing inkjets bioprinting techniques……………………………………2

SI.1 Classification of the bioprinting techniques

Figure S1 shows the classification of the existing bio-printing techniques. The new, pneumatic conveying printing, described in this manuscript is classified to droplet based printing techniques.

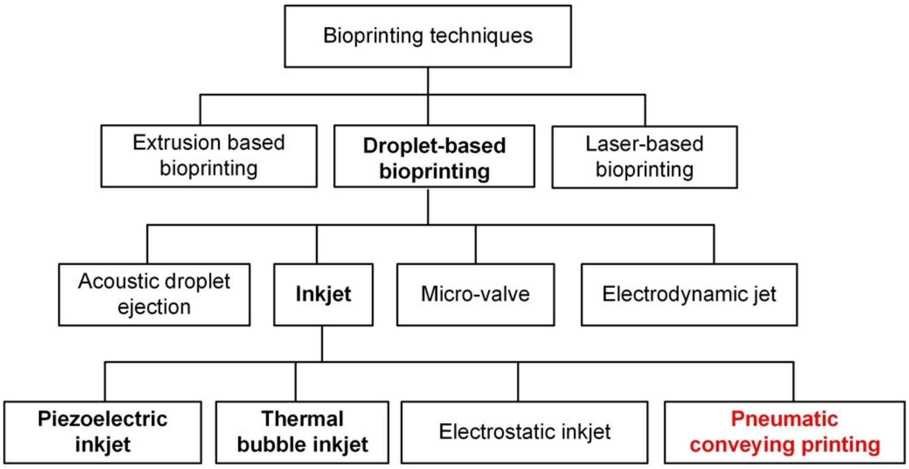

Figure S1. Classification of bioprinting techniques. Pneumatic conveying printing (PCP) is a new technique described in this manuscript.

SI.2 Limitation of existing inkjets bioprinting techniques

Figure S2 shows schematically the working principles of the two most widely used inkjet techniques, piezoelectric (Figure S2A) and thermal bubble (Figure S2B). One of the major drawbacks of inkjet bioprinting is the extremely high pressure strike in the chamber. Although previous studies[1, 2] indicated that the pressure strike is the reason for the poor cell viability, the quantitative analysis of the pressure inside the chamber is still unknown due to the difficulty of its measurement. Thanks to a computational fluid-dynamics (CFD) numerical simulation, the pressure inside the ink chamber and the shear rate at the orifice can be quantitative analyzed. Figure S2C shows the maximum pressure in the chamber and shear rate at the orifice during the ejection and in Figure S2D the same for a typical drop-ejection process. In the simulation, the density, surface tension, and viscosity of the inks were set to 1000 kg/m^3, 50 mN/m and 2 mPa·s, respectively.

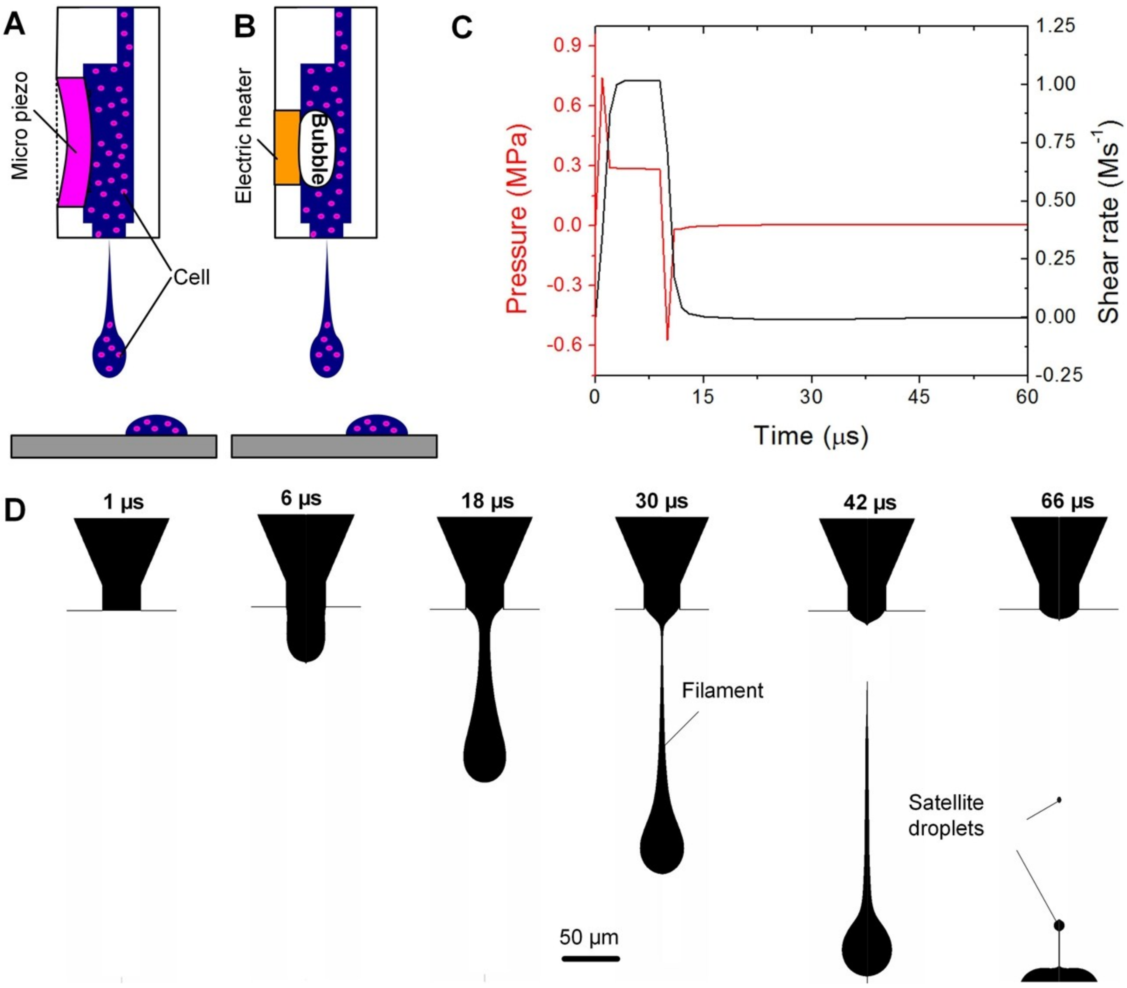

Figure S.2 Illustration of the working principles of (A) piezoelectric and (B) thermal bubble inkjet printing; (C) Pressure in the chamber and shear rate at the orifice during the ejection; (D) Typical drop ejection process of traditional inkjet printing. The density, surface tension, and viscosity of the inks were set to be 1000 kg/m^3, 50 mN/m and 2 mPa·s, respectively.

The results show that the maximum pressure occurs at the beginning of the ejection process. To detach from the orifice, the drops need to be provided with enough momentum to overcome the surface energy. For this reason, high pressures are needed in the chamber. The simulation results show that the maximum pressure can be as high as 0.7 MPa, which is 7 times the standard atmosphere pressure. It should be noted that the pressure strike is applied to the cells from the moment they enter the chamber until they are ejected. Thus all cells in

the chamber suffer such pressure strikes thousands of times, because the volume of the chamber is normally thousands of times larger than the volume of the drop. Simulations carried out for inks with a higher viscosity (20 mPa·s) show that the maximum pressure can be as high as 5 MPa to ensure ejection. The second drawback of inkjet printing arises from the use of very narrow channels, especially the inlet channel of the ink chamber, which are prone to clogging when cell-laden and viscous inks are used. The small size of the orifice also results in higher shear rates, which is another adverse impact on the cells. As shown in Figure S2C, the shear rate can be as high as 1e6 s^{-1}.

To test the performance of the traditional inkjet technique, a piezoelectric inkjet nozzle (MJ-AT-01-060) with orifice diameter 60 μm (purchased form MicroFab) was used to print our cell-laden inks (with viscosity about 30 mPa·s). Figure S3 compares of the inkjet printing performance of traditional inkjet technique with different ink viscosity.

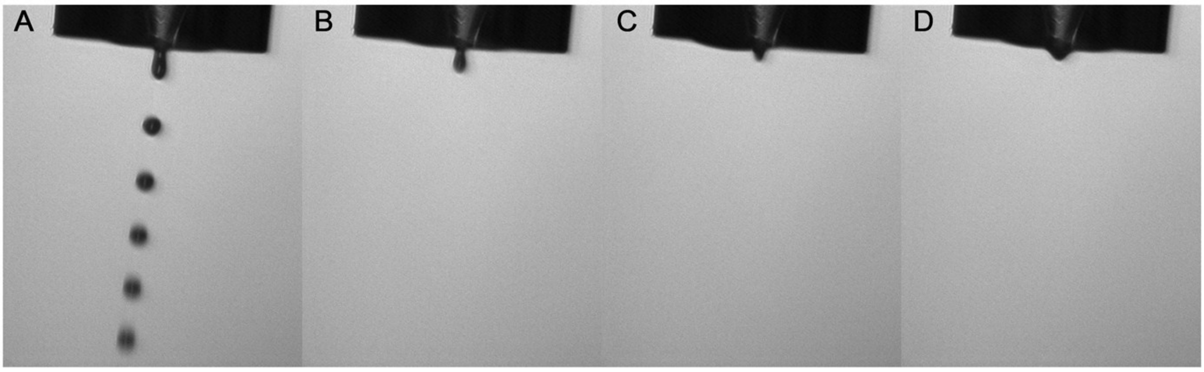

Figure S3. Comparison of the printing performance with different ink viscosity. a) viscosity 15 mPa s; b) viscosity 50 mPa s; c) viscosity 100 mPa s; d) viscosity 200 mPa s. The same waveform was applied on the nozzle. Printing frequency 1 kHz.

For inks with viscosity lower than 20 mPas, stable printing process can be achieved (Figure SI.3A). However, for the ink viscosity higher than 30 mPas, the drops cannot be detached from the nozzle. It is due to the increment in the viscous force. The drop of liquid blocks the nozzle and vibrates at the orifice (Figure SI3.B-D). This result shows clearly that traditional inject printing technique have technical limitations for the use of viscous bio-inks containing high cell loads (few millions per ml) and addition of salts, antibiotics, amino acids and other compounds required to maintain the viability of cells.

References:

1. M. S. Onses, E. Sutanto, P. M. Ferreira, A. G. Alleyne and J. A. Rogers, *Small*, 2015, **11**, 4237-4266.
2. B. Derby, *Annu. Rev. Mater. Res.*, 2010, **40**, 395-414.
